# Crowdsourcing Precision Cerebrovascular Health: Imaging and Cloud Seeding A Million Brains Initiative™

**DOI:** 10.3389/fmed.2016.00062

**Published:** 2016-11-22

**Authors:** David S. Liebeskind

**Affiliations:** ^1^Department of Neurology, Neurovascular Imaging Research Core and UCLA Stroke Center, University of California Los Angeles, Los Angeles, CA, USA

**Keywords:** stroke, imaging, data science, precision medicine

## Abstract

Crowdsourcing, an unorthodox approach in medicine, creates an unusual paradigm to study precision cerebrovascular health, eliminating the relative isolation and non-standardized nature of current imaging data infrastructure, while shifting emphasis to the astounding capacity of big data in the cloud. This perspective envisions the use of imaging data of the brain and vessels to orient and seed A Million Brains Initiative™ that may leapfrog incremental advances in stroke and rapidly provide useful data to the sizable population around the globe prone to the devastating effects of stroke and vascular substrates of dementia. Despite such variability in the type of data available and other limitations, the data hierarchy logically starts with imaging and can be enriched with almost endless types and amounts of other clinical and biological data. Crowdsourcing allows an individual to contribute to aggregated data on a population, while preserving their right to specific information about their own brain health. The cloud now offers endless storage, computing prowess, and neuroimaging applications for postprocessing that is searchable and scalable. Collective expertise is a windfall of the crowd in the cloud and particularly valuable in an area such as cerebrovascular health. The rise of precision medicine, rapidly evolving technological capabilities of cloud computing and the global imperative to limit the public health impact of cerebrovascular disease converge in the imaging of A Million Brains Initiative™. Crowdsourcing secure data on brain health may provide ultimate generalizability, enable focused analyses, facilitate clinical practice, and accelerate research efforts.

## Introduction

Precision medicine and the concept of theranostics are based on leveraging data about an individual in specific context to optimize health outcomes (1). This individualized, contextual approach to data boldly contrasts with the traditional framework of medical evidence that emanates from randomized, controlled, or population-based clinical trials. Clinical trials are an important vehicle to study selected, hypothesis-driven approaches about diagnosis or therapy of particular disorders, prudently defining inclusion of certain subjects and measuring key variables of interest. For the incredibly common, yet complex topic of cerebrovascular health, the use of somewhat arbitrary definitions and imperative to control for myriad factors may skew the ultimate impact of research efforts. Research on cerebrovascular health overwhelmingly emphasizes control of data diversity, rather than consideration of the complex relationships between numerous variables. In our attempts to determine what health-care strategy is right for a particular individual at a specific moment, we exclude the majority of susceptible individuals from the study population, focus only on dramatic or significant changes in disease magnitude, and often fail to account for many other causes that impact long-term health outcomes (2). Crowdsourcing, an unorthodox approach to modern data collection in medicine, creates an unusual paradigm to study precision cerebrovascular health, eliminating the relative isolation and non-standardized nature of current imaging data infrastructure, while shifting emphasis to the astounding capacity of big data in the cloud. This perspective envisions the use of imaging data of the brain and vessels to orient and seed A Million Brains Initiative™ that may leapfrog incremental advances in stroke and rapidly provide useful data to the sizable population around the globe prone to the devastating effects of stroke and vascular substrates of dementia.

A consideration of crowdsourcing data on cerebrovascular health requires discussion on the types, longitudinal or temporal changes, distribution, logistics, ownership, sharing, and leveraging of data that are relevant to stroke and neurovascular disorders. Current technology and the digital revolution in electronic health records, secure cloud-based resources, cloud computing, and metadata may be used to transform readily available imaging and other data that millions of individuals may share and learn about how their brain health changes over time. This novel approach simultaneously avoids the extreme inefficiencies in current medical care of cerebrovascular health. Imaging of the brain and vessels is essential in characterizing the phenotype of stroke or cerebrovascular health, providing a scaffold to build other data dimensions. Clinical expertise in this highly specialized subspecialty is also crucial, yet easily adapted to this large-scale electronic format. It remains to be delineated, exactly how such expertise would be vetted. Crowdsourcing secure data on brain health may provide ultimate generalizability, enable focused analyses, facilitate clinical practice, and accelerate research efforts.

## Imaging Definitions

Definitions are key in discerning precision health strategies, individualizing care, and quantifying the impact of any particular factor on brain integrity. Cerebrovascular health or status of an individual’s brain relating to the blood vessels or blood flow is relatively neglected in contrast to defining stroke symptoms and particular treatment approaches. The episodic nature of stroke or the discrete period when symptoms may affect an individual due to vascular events in the brain slants our definition and attention away from health to focus on acute disease. As a result, asymptomatic disorders or silent stroke and more chronic or insidious processes evade detection until imaging is acquired. Many individuals undergo brain imaging for other reasons and are then noted to have silent strokes or blood vessel changes. Imaging is often used to define acute from subacute cerebrovascular changes in the brain, and the type of stroke is most commonly defined by imaging patterns. Imaging may be used to define the success of certain therapies, as with recanalization or reperfusion of the ischemic brain with thrombolysis or thrombectomy in acute stroke. Alternatively, the chronic impact of microvascular disorders and risk factor modification or response to certain treatments may be monitored with serial imaging acquired at different time points. The severity and extent of cerebrovascular changes are described with imaging measures such as narrowing of a vessel, lesion size, or brain atrophy. Quantitative analyses of raw imaging data are therefore instrumental, without imparting the bias of a particular reader or observer. Raw imaging data of stroke phenotype or incidental findings can therefore define the presence, extent, and temporal evolution of cerebrovascular health.

## Data

Imaging is fundamental, yet numerous sources and types of data on cerebrovascular health may be used to define an individual. When imaging is absent, it remains difficult to ascertain the extent of any cerebrovascular disease. In contrast, the amount of other data types may vary depending on context. For instance, clinical or other biological data may be minimal if someone undergoes an MRI after an initial complaint of headaches. In other scenarios, detailed and serial measures of blood biochemistry and other functional biological data may be acquired. Even the most rudimentary examination data such as blood pressure values or basic historical elements may vary depending on context of where and when an individual is evaluated. Despite such variability in the type of data available, the data hierarchy logically starts with imaging and can be enriched with almost endless types and amounts of other clinical and biological data (3).

The location, availability, and access to such data are a separate consideration. In clinical trials, only a subset of the available clinical and biological data is abstracted in a case report form. In the past, even the imaging characterization based on actual data was inferred rather than directly evaluated from source datasets. The structure of clinical trials inevitably requires resource allocation and costs that must weigh the value of particular data types. When a clinical trial is focused on the therapeutic effect of an endovascular device for stroke, only select interventional variables may be collected while ignoring other data types. This does not mean that individual pathophysiology or data relating to specific patients or contexts are inconsequential. In fact, many of these data types are critical for generalizability and generating real world evidence that is recently the focus of regulatory agencies. Most commonly, the majority of data on an individual’s cerebrovascular status are isolated or contained within a medical facility, with limited access beyond the initial period when such data are obtained. Data are siloed and infrequently used for comparison sake, especially when individuals have care or imaging acquired across numerous health-care facilities. Such data that are highly specific to an individual are thereby scattered, causing redundancy and inefficiency. The most logical focal point for any type of data on cerebrovascular health is, therefore, with the patient or individual, wherever they go, over time. Increasingly, people are securely storing their medical imaging and other health data online in the cloud (4). As mobile health technology evolves, many other types of health data, such as physical activity and even physiological parameters are stored in this fashion. This is ultimately the most logical location and access point for any individual to store, chronicle, and manage data about their brain health. Requesting permission for a copy of imaging from the film library at the local hospital or outpatient imaging facility, accessing these images, and making them available to others have been revolutionized with cloud-based providers. Importantly, such storage and access may be preserved indefinitely.

Ownership, sharing, and leveraging of data on cerebrovascular health rest with the individual, not an external entity such as a facility, health-care organization, sponsor, or governmental agency. Crowdsourcing allows an individual to contribute to aggregated data on a population, while preserving their right to specific information about their own brain health. The use of anonymized or de-identified data may be used for an individual to store their own brain imaging, compare brain health with population-derived data, access a variety of computing modules, and track how these things change over decades (5). Cloud seeding or sharing of imaging with other data may be encouraged through the crowdsourcing model of social media and the Internet. Similar to any online search engine in the future, one may literally use their own brain to seek answers about cerebrovascular health. The cloud now offers endless storage, computing prowess, and neuroimaging applications for postprocessing that is searchable and scalable (6). Most individuals, however, do not realize that they may own their own brain imaging data in such fashion. The rapidly expanding potential virtual library of brain imaging in the cloud has almost endless dimensions. Comparison of serial imaging acquired at multiple time points over years and institutions may serve as a virtual global PACS, much like a universal electronic health record (4). In stroke or cerebrovascular health, the profound focus on imaging suggests that this is the most logical axis to pursue. Rather than the impression of a single imaging reader, one may obtain innumerous measures and opinions on the imaging data that never degrade with time.

## Collective Expertise in Stroke

Collective expertise is a windfall of the crowd in the cloud and particularly valuable in an area such as cerebrovascular health. The complex nature of stroke, multidisciplinary aspects, and imaging considerations now demand input from a diverse source of experts. Modern technology has already been leveraged in this manner for the delivery of acute stroke telemedicine, where expertise can be applied around the globe to streamline definitive therapies for cerebrovascular disease. Amassing data alone are insufficient without expert involvement, and in many scenarios, there are no theoretical limits on the amount of expertise available. In other disorders, clinical consultations in the cloud may be maximized with examples such as CrowdMed (7). However, stroke expertise is essential. Creating an endless volume of brain imaging data without the focus on cerebrovascular health or clinical impact is a recipe for erroneous data exploration. Even institutional informatic initiatives to harness all brain imaging are pointless without cerebrovascular expertise. Ideally, crowdsourcing across wide geographical areas and disparate environments allows for collective expertise in stroke that is generalizable and simultaneously rooted in detailed individual data.

## A Million Brains Initiative™

The rise of precision medicine, rapidly evolving technological capabilities of cloud computing, and the global imperative to limit the public health impact of cerebrovascular disease converge in the imaging of A Million Brains Initiative™. A Million Hearts^®^ seeks to address stroke and cardiovascular disease, yet this cannot be achieved without imaging the brain, where stroke occurs (8). Collective expertise and access to the secure, raw data on the brain and vessels of a million individuals is not just feasible, but tenable with current technology paired with the incentives of a crowdsourcing model. Amassing such a dataset cannot be sponsored by any individual entity, whereas the costs and need to acquire imaging are obviated with the use of existing data. The inefficiencies in current neuroimaging practice may be addressed with increasing participation in cloud-based storage that is fully HIPAA compliant to ensure highest standards for individual privacy. Cloud seeding, however, is only an initial step far short of maximally utilizing current technology and online collective expertise.

Cloud seeding the existing imaging datasets by a million individuals would provide secure storage, indefinitely, from anywhere around the world. The standardized DICOM file structure of medical images and the extensive panoply of variables germane to cerebrovascular disease in the Common Data Elements (CDE) enable a searchable and scalable platform for crowdsourcing (9). Individuals may be incentivized by the need for secure storage and the ability to readily view their images without the need for a DICOM viewer on a local computer. Only a fraction of the public is currently aware of their right to access and obtain their medical images. A neuroimaging campaign to underscore this health-care provision would allow for crowdsourcing of secure or anonymized datasets, digitally preserved in the cloud. Numerous providers currently enable individuals to do this, manipulate, postprocess, measure, graphically display, and even print a 3D replica of their own brain and blood vessels. Sharing datasets would eliminate the need for repeated scanning across medical institutions simply because of current accessibility issues (10). This provides tremendous advantages for the individual over the isolated goal of imaging from a business perspective. The crowdsourcing model of big data would allow for age- and sex-matched comparisons on numerous metrics of brain morphology and even function. High-performance computing algorithms already have the ability to process cloud-based imaging, and future modules could be sequentially deployed to provide enormous information on the cerebrovascular status of individuals within a truly global population (5, 6, 11). Unlike other neuroimaging initiatives, such a vision is fueled by individuals, volunteering and yet, immediately benefiting from the novel information resource simultaneously created. Seeding the cloud with brain images is no different than current online searches or blogs where the end user automatically serves as a contributor. Individuals may see their own brain, utilize the raw data to calculate standard quantitative measures such as cortical thickness, depict changes over time, avoid the relatively subjective impressions currently employed, and leverage the ability to tap clinical expertise and endless virtual consultants around the world. Conveniently, the individual imaging and paired clinical data can be securely shared and controlled with care providers, while the aggregated population data contribute to scientific advancement. Importantly, this structure overcomes the current impasse on data sharing and ownership that limits the public health impact of clinical research (12). Such a crowdsourcing model to power precision cerebrovascular health on a large scale preserves the role and separate function of randomized trials, yet the cloud is a powerful vehicle and incredibly rich data source for observational studies.

The infrastructure and logistics of A Million Brains Initiative™ use imaging as a backbone, capitalizing on the overwhelming abundance of existing data, and essential role in defining cerebrovascular health. The goal of a million individuals is symbolic, yet easily achieved if one accounts for the volume of neuroimaging acquired on a daily basis and the relatively modest storage demands of such data. One million CT or MRI brain imaging studies and associated data can be managed in terabytes, an incredibly small fraction of the cloud. The organization and productivity of such data may leverage the expertise and collective input of stroke imagers around the world. The clinical and broader public health impact would benefit from precision data mining techniques, machine learning, and additive computational modules. Seeding the cloud with a million brains to study stroke and cerebrovascular health efficiently multiplies sample sizes and enables endless statistical modeling initiated by individuals motivated to learn more about their own brains. Limitations, undoubtedly, exist, including acceptance of the idea of uploading an individual’s brain imaging and clinical data to a repository and cultural differences in this regard, issues with variability in quality of imaging data and in clinical data, validity of non-imaging data to be collected, potential challenges in involving enough experts to handle a massive amount of data, and potentials and pitfalls of involving non-experts in image analysis or data classification tasks.

## Conclusion

Crowdsourcing the existing imaging and other data of a million individuals is a realistic goal of defining precision cerebrovascular health in the modern digital era. Imaging of the brain and blood vessels provides an intuitive and fundamental architecture of a potentially vast data source for individuals, clinicians, and scientists. An evolving and modular structure securely leverages technological advances and simplifies current barriers in health-care data to provide data that are big on dimension, depth, and individual details.

## Author Contributions

DL conceived, designed, drafted, and granted final approval for this manuscript. DL agreed to be accountable for all aspects of the work in ensuring that questions related to the accuracy or integrity of any part of the work are appropriately investigated and resolved.

## Conflict of Interest Statement

The author declares that this manuscript was developed in the absence of any commercial or financial relationships that could be construed as a potential conflict of interest.
